# A retrospective cohort study of premature neonatal mortality rates and contributing factors in a tertiary referral NICU in Addis Ababa, Ethiopia from 2022 to 2023

**DOI:** 10.1016/j.cegh.2025.102118

**Published:** 2025-07-07

**Authors:** Gregory C. Valentine, Krystle M. Perez, Olivia C. Brandon, Sharla Rent, Gal Barbut, Merhawit Abadi, Gesit Metaferia, Redeat Workneh, Mahlet Abayneh

**Affiliations:** a Department of Pediatrics, Division of Neonatology at University of Washington, Seattle, WA, USA; b Department of Obstetrics & Gynecology, Baylor College of Medicine, Houston, TX, USA; c Department of Pediatrics, Division of Neonatology, Duke University, Durham, NC, USA; d Duke Global Health Institute, Duke University, Durham, NC, USA; e Department of Pediatrics, Division of Neonatology, University of Rochester, Rochester, NY, USA; f Department of Pediatrics and Child Health, Division of Neonatology, St. Paul’s Hospital Millennium Medical College, Addis Ababa, Ethiopia

**Keywords:** Ethiopia, Neonatal mortality, Global health, Premature outcomes

## Abstract

**Background:**

Prematurity remains the leading contributor for neonatal death globally, with Ethiopia having the 4th highest incidence of neonatal deaths. Clarifying specific contributors of prematurity-related neonatal death is critical to improve outcomes.

**Objective:**

We evaluated neonatal causes of death among premature neonates at St. Paul’s Hospital Millennium Medical College’s (SPHMMC) neonatal intensive care unit (NICU).

**Study design:**

Through a retrospective electronic database, we evaluated mortality outcomes for neonates <37 weeks’ gestation admitted to SPHMMC NICU from February 2022–May 2023, excluding those with congenital anomalies. Logistic regression models assessed the association between variables and outcomes.

**Results:**

Of 1033 premature neonates included in the analysis, 228 (22.1 %) died. Mortality was inversely related to birthweight with extremely low birthweight neonates (≤1000g) having 84 % mortality. The top 3 causes of prematurity-related mortality included respiratory distress syndrome (67.7 %, 159/228), sepsis (39.5 %, 90/228), and pulmonary hemorrhage (25.0 %, 57/228). Neonates that died from pulmonary hemorrhage had higher antenatal corticosteroids (ACS) exposure (66.7 % vs 36.7 %, p < 0.001), less chorioamnionitis (29.5 % vs 55.5 %, p = 0.004), and higher cesarean section delivery (71.9 % vs 53.1 %, p = 0.02). Notably, there were no associations between mortality and ACS exposure or lack thereof in the overall cohort in multivariate models.

**Conclusion:**

While respiratory distress syndrome and sepsis are commonly reported findings of premature mortality, surprisingly, 25 % of premature newborns died from pulmonary hemorrhage at SPHMMC NICU. Further investigations are needed to clarify the causes and contributing factors leading to premature death and to determine whether pulmonary hemorrhage is an underreported, underrecognized contributor to prematurity-related mortality globally.

## 1. Introduction

Prematurity, defined as delivery before 37 weeks’ gestation, is the primary contributor to both neonatal mortality as well as overall under-5 mortality worldwide.^1,2^ More than 60 % of all preterm births occur in low- and middle-income countries (LMICs), with 28.7 % occurring in sub-Saharan Africa.^3,4^ Notably, 98 % of all neonatal deaths occur in LMICs.^1,2,4,5^ While overall under-5 mortality is improving due to advancements in the treatment of other common childhood illnesses and diseases, neonatal mortality has only marginally improved.^4^ With minimal improvements in the incidence of preterm birth over the past three decades worldwide,^3^ the burden of prematurity as an etiology for neonatal mortality continues to grow with each passing year. Thus, efforts are needed to evaluate specific causes and contributors to prematurity-related mortality in LMICs, especially sub-Saharan Africa, to assess and plan interventions to improve neonatal, infant, and pediatric outcomes.^3^

Ethiopia, a landlocked country in East Africa, is among the top five countries worldwide with the highest number of preterm births.^3^ In Ethiopia, the preterm birth rate of ~12.9 % has remained largely unchanged from 2010 through 2020 per recent analyze seven amidst an expanding population.^3^ Ethiopia’s preterm birth rate trend is similar to overall unchanged trends in the larger sub-Saharan region, with an estimated preterm birth rate consistently at 10.1 % from 2010 to 2020.^3^ Saint Paul’s Hospital Millennium Medical College (SPHMMC) is the second largest hospital in Ethiopia and is a central, tertiary-referral hospital in Ethiopia’s capital - Addis Ababa. SPHMMC serves a population of approximately 5 million people, and the labor and delivery units routinely have more than 12,000 deliveries per year.^6^ The SPHMMC neonatal intensive care unit (NICU) has over 60 beds and is served by 2 neonatologists, 4 neonatal-perinatal fellows, resident physicians, clinical officers, medical students, nursing, and ancillary staff. Similar to other resource limited NICUs, electronic medical records are not available or routinely used in the SPHMMC NICU, making continuous evaluation and monitoring of patterns and/or contributors to neonatal mortality and morbidity challenging. Nonetheless, SPHMMC has had long-standing commitment to improving neonatal outcomes and quality improvement, evidenced by their active roles in the Ethiopian and African Neonatal Networks, and joining as one of the first LMIC members to the Vermont Oxford Network in 2018.^7^ Although these networks facilitate monthly review of outcomes, the databases do not provide specific granular information pertaining to these outcomes. Our primary aim was to assess and delineate the causes of death as documented by the physicians at the time of death. We hypothesized that the most common causes of death of premature newborns would be respiratory distress syndrome, sepsis, and asphyxia. Our secondary aims were to (a) assess differences in mortality based on birthweight using increments of 100 g, (b) determine whether differences existed in mortality rates based on time since birth, (c) evaluate whether mortality rates fluctuated by month, and (d) measure whether antenatal corticosteroid exposure altered the risk of mortality among premature neonates at SPHMMC. We hypothesized that mortality would increase for smaller birthweight babies, that mortality would be highest in the first 3 days after birth, mortality would be relatively similar aross all months, and antenatal corticosteroid exposure would reduce the risk of mortality.

## 2. Materials and methods

We performed a retrospective cohort study and included premature neonates (<28 days of life) born <37 weeks’ gestation admitted to the SPHMMC NICU from February 2022–May 2023. In partnership with SPHMMC and following IRB approval at both SPHMMC and the University of Washington, an electronic database was created to record specific prematurity-related causes of death at SPHMMC according to clinical documentation by physicians. The database consists of 1080 variables, including demographic and clinical data from every premature neonate admitted to the SPHMMC NICU. Included neonates were born <37 weeks’ gestation by best known obstetric dating, including first trimester ultrasound if available. A data assistant and data supervisor were trained over the course of a 6-week period on the use of the online, encrypted, password-protected, Research Electronic Data Capture (REDCap) database. To reduce any bias and inaccuracies, chart data extraction went through a quality assurance process, which included training on extraction of files from the paper charts to the REDCap database, identifying the necessary abstraction variables from the paper charts, correctly entering the data into the REDCap database in a de-identified manner, and intermittent audits performed by the data supervisor. Specifically, audits and quality control checks were performed daily for the first month of the database development and implementation gradually progressing to biweekly and then monthly audits including review of five percent of randomly selected charts. Additional evaluations were performed via biweekly audits by the study PI of the online REDCap database and discussed with the study team to review individual charts that had outliers within their data entry.

Data and status updates on the database were reviewed by the study team on a biweekly basis throughout the entirety of the study period. Information and data collected included the following: maternal data including comorbidities during pregnancy, singleton or multiple gestation, neonatal admission weight, Apgar scores, daily fluid intake over the first 14 days (including enteral and parenteral feed or fluid contributions), and daily weights over the first 14 days, among other key variables.

Patient charts were inputted into the database at the time of discharge, death, transfer to another facility or family decision to leave against medical advice. All data was de-identified, and a waiver of informed consent was obtained and approved by the SPHMMC and UW IRBs which both approved this study.

The inclusion criteria for this study were: (a) premature newborn as defined as <37 weeks’ gestation by best obstetric or neonatal estimate, (b) receiving care in the SPHMMC NICU. The exclusion criteria were: (a) any known congenital or genetic anomaly at the time of admission to the SPHMMC NICU, (b) readmission hospitalizations were not included, (c) missing gestational age, and (d) missing information on whether the newborn died during initial hospitalization.

For the purposes of this manuscript, we have used the term “neonate” or “newborn” to encompass babies born and admitted to the SPHMMC and who died during this hospitalization in the NICU. While neonate generally is defined as the first 28 days after birth, as >95 % of the babies included in this study died within the first 28 days. However, <3 % died after 28 days.

### 2.1. Causes of death- clinical outcomes

Mortality was based on all-cause mortality during hospitalization. For neonates who died, primary cause of death was extracted from the neonate’s medical record paper chart as documented by the physician with non-exclusive entry as to additional causes of prematurity-related death. Pulmonary hemorrhage was defined clinically as blood in the endotracheal tube or coming from the oropharynx.

### 2.2. Study size

As our primary analysis was to evaluate the overall causes of mortality among premature neonates which is descriptive in nature, a power calculation was not determined to be required for such an analysis. Instead, we evaluated outcomes over approximately 1 year to provide an entire year of data to offset any potential seasonal or other variations that may occur month-to-month.

### 2.3. Statistical analysis

Fisher’s exact test and Mann Whitney U-tests were used to compare groups in terms of categorical and numerical variables, respectively. Mortality rates were further assessed by birthweight category (100-g increments until >2000 g), by chronological age categories (<24 h, 24–72 h, >72 h to 7 days after birth, and >7 days after birth), and by moving-windows monthly basis correlating to monthly NICU admission rates. Due to limited overall numbers of neonates with birthweights <1000 g, all neonates with birthweights <1000 g were grouped together for the sub-analysis by birthweight categories. Analyses requiring accurate prenatal data, including antenatal corticosteroids (ACS), were limited to inborn neonates. Logistic regression models were used to examine the association of ACS with mortality. R was used for data analysis (version 4.3.2, R Foundation for Statistical Computing, Vienna, Austria). Both R and Prism were used for data visualization (version 10.1.1, GraphPad Software, San Diego, CA, USA). A probability level less than 0.05 was considered significant. The strengthening the reporting of observational studies in epidemiology (STROBE) checklist was used in developing this manuscript (see supplemental material).^8–10^

## 3. Results

### 3.1. Overall mortality rate and etiology

A total of 1379 neonates (including both term and preterm newborns) were admitted to the SPHMMC NICU during the study period of February 9th, 2022–May 9th, 2023. After excluding n = 13 with missing gestational ages at birth, n = 80 with congenital anomalies, n = 244 who were term, and n = 9 with unknown mortality outcome, 1033 premature neonates were included in the study analyses. A total of 228 of these neonates died during hospitalization, representing an overall mortality rate of 22.1 %. Characteristics of those who died versus those who survived are presented in Table 1. The leading causes of mortality were: respiratory distress syndrome 69.7 % (159/228), presumed or culture-positive sepsis 39.5 % (90/228), and pulmonary hemorrhage 25.0 % (57/228), see Supplemental Table 1.

### 3.2. Mortality by birthweight category

**Fig. 1.**
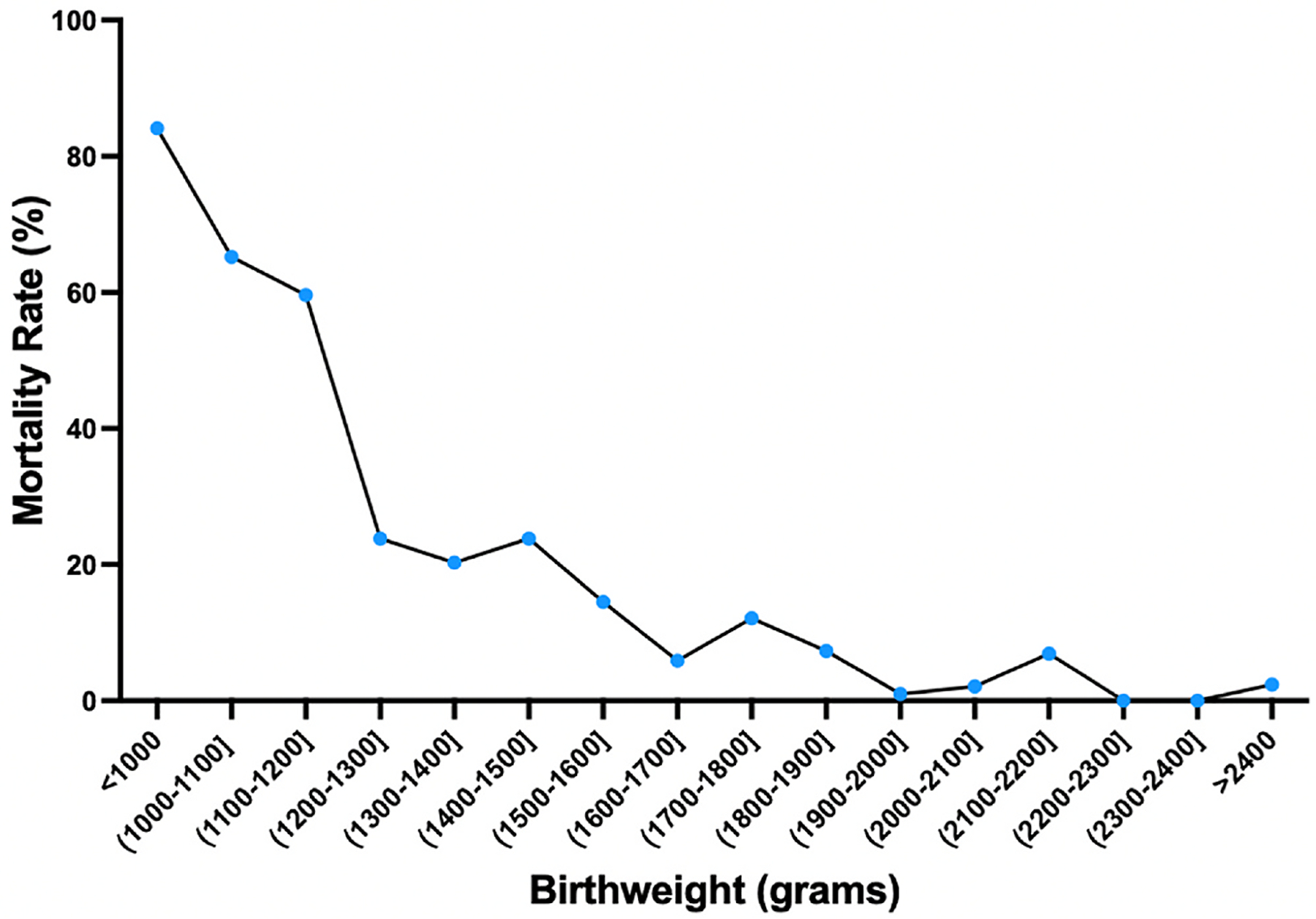
Mortality rates by birthweight category. 38 babies missing birthweight information.

Forty neonates had missing birthweight data and were not included in the subgroup analysis by birthweight. Mortality was inversely related to birthweight (Fig. 1, unadjusted analyses see Supplemental Fig. 1). The group with the highest mortality was in neonates with a birthweight of <1000 g who had an 84.1 % mortality rate (74/88). Overall, the mortality rates for neonates with birthweights between 1001 and 1200 g was 62.1 % (64/103), 1201–1500 was 22.8 % (46/202), 1501–2000g 7.3 % (26/356), >2000g was 3.3 % (8/244). The overall mortality rate for very low birthweight (VLBW; defined as birthweight <1500 g) neonates was 46.8 % (184/393).

### 3.3. Chronological age at time of death

Of the 228 total deaths recorded during the study period, 13 patients (5.7 %) had missing data specifying the date of death. Therefore, the remaining 215 premature neonates were included in this sub-analysis. Twenty-six (12.1 %) died within the first 24 h of birth, 88 (40.9 %) patients died between 24 and 72 h after birth, 54 (25.1 %) died between 72 hours and 1 week of age, and 47 (21.9 %) died >1 week of age (Supplemental Fig. 2, Supplemental Table 2).

**Fig. 2.**
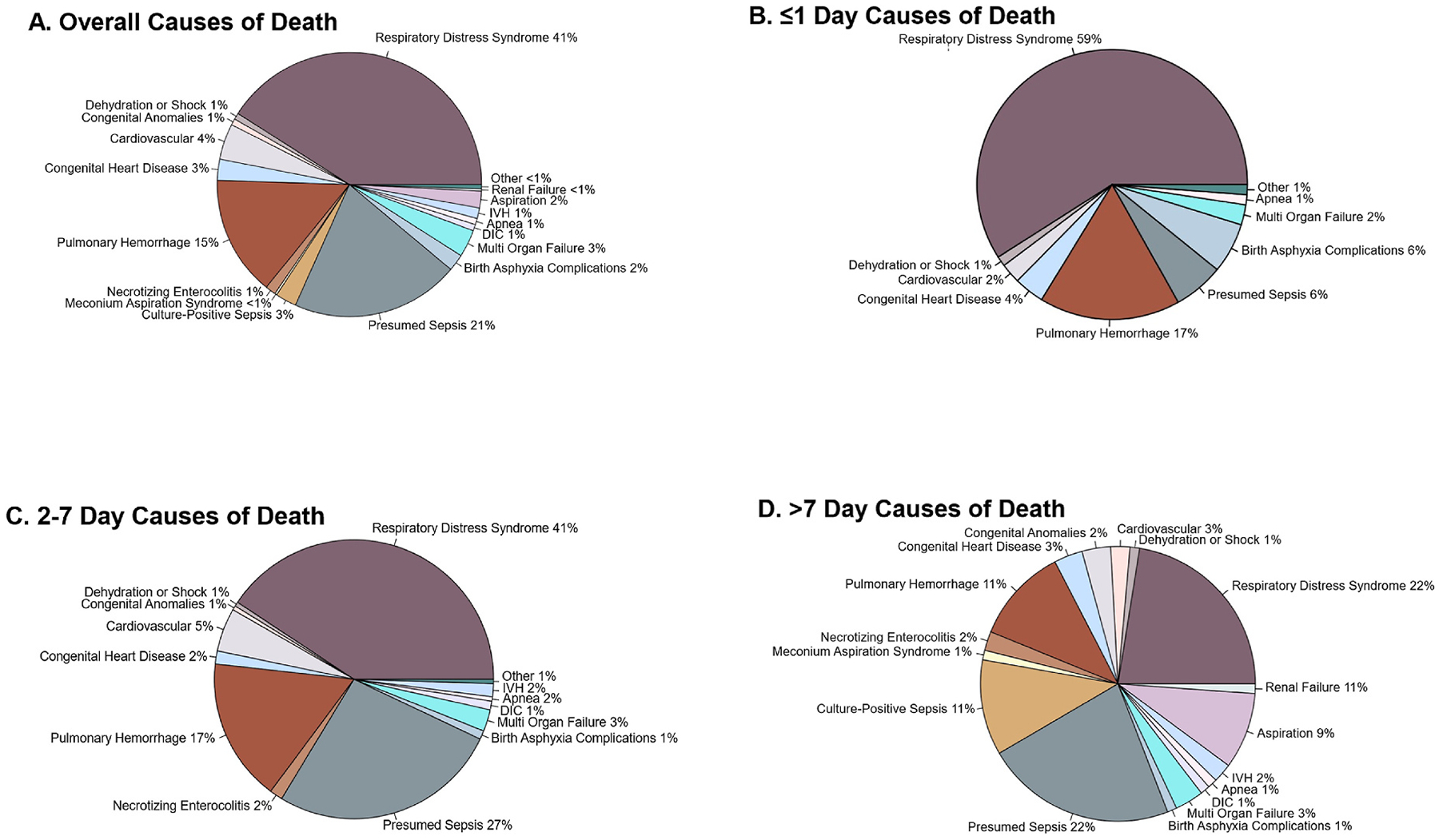
Breakdown of causes of death among all babies who died by overall cause of death (A), ≤1 day (B), between 1 and 7 days (C), and after 7 days (D). Of note, the percentages of the causes of death are depicted as a percentage of all possible causes of death. To see the breakdown per patient that died, please see Supplemental Table 1.

The causes of neonatal mortality are shown in Fig. 2 and Supplemental Fig. 3. Within the first 24 h after birth, the top three causes of mortality among premature neonates were: respiratory distress syndrome 80.3 % (49/61), pulmonary hemorrhage 23.0 % (14/61), and presumed or culture-positive sepsis 8.2 % (5/61), see Supplemental Table 1. Between days 2 and 7 days after birth, the top three causes of mortality among premature neonates were: respiratory distress syndrome 75.7 % (81/107), presumed or culture-positive sepsis 49.5 % (53/107), and pulmonary hemorrhage 30.8 % (33/107), see Supplemental Table 1. After the first week after birth, the top three causes of mortality among premature neonates were: presumed or culture-positive sepsis 63.8 % (30/47), respiratory distress syndrome 42.6 % (20/47), and pulmonary hemorrhage 21.2 % (10/47), see Supplemental Table 1. Of note, percentages depicted in Fig. 2 are based on number of cases divided by total causes of death (in which a neonate may have more than 1 cause of death) whereas percentages depicted in Supplemental Table 1 are based on number of cases over all cases of mortality.

### 3.4. Monthly mortality rate & association with monthly admission rates

We further evaluated mortality based on a monthly basis to assess variation in overall premature mortality rates and admission rates to the SPHMMC NICU across a 12-month period. The monthly admission rate was lowest in April 2023 with admission of 30 premature newborns and was highest in December 2022 with admission of 83 premature newborns. The overall monthly mean admission rate was 67 premature neonates per month. The highest mortality rate occurred in June 2022 with a rate of 32.3 % (21 deaths/65 admissions); the lowest mortality rate occurred in October 2022 with a rate of 10.8 % (7 deaths/65 admissions).

### 3.5. Mortality & association with antenatal corticosteroid exposure

Of the 1033 neonates included, 11 did not have available information on ACS exposure and were not included in this sub-analysis. Of the 1022 premature neonates included, 52.9 % (541/1022) were exposed to ACS with 37.2 % (n = 380) exposed to a full, 4-dose course of dexamethasone (the Ethiopian recommended ACS), see Supplemental Fig. 4. Data related to exposure to ACS were also evaluated for differences related to maternal and neonatal characteristics, including sex, multiple births, mean birthweight, Apgar score at 1 and 5 min, urban versus rural delivery, and maternal HIV status (Table 2). In an unadjusted logistic model, exposure to ACS was associated with significantly increased odds of mortality (unadjusted odds ratio 1.61,95 % CI 1.14, 2.28) with additional differences in comparing raw mortality rates among neonates with ACS exposure compared to those without any ACS exposure (139/541, 257.7 % vs 86/481, 17.9 % mortality, p = 0.003). However, after controlling for the variables with significant differences between those with ACS exposure (gestational age, intrauterine growth restriction (IUGR) status, mode of delivery, pre-eclampsia, multiple gestation, and requirement for any respiratory support during hospitalization), premature neonates exposed to ACS did *not* have a difference in mortality compared to neonates who were unexposed (adjusted OR = 1.09, 95 % CI 0.65–1.84, p = 0.75).

When evaluating the relationship between in-hospital mortality and full ACS course (4+ doses of dexamethasone), partial ACS course (1–3 doses of dexamethasone), or lack of any ACS exposure, the only significant finding was in the unadjusted analyses with a full course of steroids showing a significantly higher odds of mortality (OR 1.69, 95 % CI 1.17, 2.45, p = 0.005). However, this was no longer the case after adjustments (aOR 1.19, 95 % CI 0.68, 2.07, p = 0.54), see Supplemental Table 3.

### 3.6. Evaluation of factors related to mortality attributed to pulmonary hemorrhage

Given the high prevalence of pulmonary hemorrhage, we further assessed factors that were associated with premature neonates who died from pulmonary hemorrhage compared to neonates who died from other causes (Table 3).Significant associated factors among premature neonates who died from pulmonary hemorrhage included: higher exposure to ACS (66.7 % vs 36.7 %, p < 0.001); less exposure to chorioamnionitis (29.5 % vs 55.5 %, p = 0.004), and delivery via cesarean section (71.9 % vs 53.1 %, p = 0.02). Neonates that had pulmonary hemorrhage listed as a cause of death did not have significant differences in respiratory support needs, gestational age, birthweight, or any other evaluated neonatal factors compared to babies without pulmonary hemorrhage listed as a cause of death. When pulmonary hemorrhage was listed as a cause of death, the top three co-documented contributors to mortality were RDS, prematurity, and presumed sepsis as cause of death was not exclusive to a singular cause (see Supplemental Table 4).

## 4. Discussion

### 4.1. Principal findings

In the period of February 2022–May 2023, the overall preterm mortality rate within the SPHMMC NICU was 22.1 %. Mortality was inversely related to birthweight with the highest mortality rate of 84.1 % among ELBWs. The period with the largest burden of mortality was between 24 and 72 h after birth accounting for more than 40 % of all prematurity-related deaths in the SPHMMC NICU. Utilization of antenatal corticosteroids did not significantly impact the mortality rate after adjusting for important covariates. The leading causes of death were RDS, sepsis (culture proven or presumed), and pulmonary hemorrhage. The finding of pulmonary hemorrhage within the top three etiologies of preterm mortality was unexpected with limited publications describing pulmonary hemorrhage as a common contributor to prematurity-related mortality within LMICs.

### 4.2. Clinical implications

Prematurity is the leading cause of neonatal mortality and associated with significantly increased risk of neurodevelopmental disabilities and higher lifetime healthcare expenses.^3,11–13^ Yet, the global burden of preterm birth has not changed in the past decade.^3^ Preterm birth disproportionately burdens low- and middle-income countries (LMICs) as over 55 % of all live births occur in southern Asia and sub-Saharan Africa, with these regions representing 65 % of all preterm births.^3^ Furthermore, as increasing proportions of births are occurring in healthcare facilities(as opposed to home births) these facilities require specialized training and resources to care for small and/or sick newborns (SSN).^14,15^ Recognizing this public health crisis, the World Health Organization has convened global neonatal and perinatal experts seeking to improve the quality of care for SSNs, especially those being treated in facilities in LMICs. These efforts complement the Every Newborn Action Plan, launched in 2014, that prioritized 10 core indicators to measure maternal and neonatal outcomes, including neonatal mortality rate, essential newborn care, and ACS use, among others.^16^

The findings in our study demonstrate consistent patterns of mortality rates among premature infants as have been previously reported. Specific to Ethiopia, a prior publication shared mortality rates from 2016 to 2018 across 5 tertiary hospitals in Ethiopia, including SPHMMC, and reported an overall prematurity-related mortality rate of 22.7 %.^17^ Our present study, conducted in 2022–2023 reports a similar overall preterm mortality rate of 22.1 % at SPHMMC NICU. Additionally, extremely low birthweight (ELBW; <1000-g birthweight) neonates had the highest mortality rate in this cohort at 84 %, also consistent with a 2021 systematic review and meta-analysis that highlighted the variation in ELBW mortality within and between various categories of low- and middle income countries; in the study, ELBW mortality rates were estimated at 82 % in low-income (including Ethiopia), 72 % in lower middle-income, and 61 % mortality in upper middle-income countries.^18^ Comparably, the EBLW mortality rate has been reported to be 9.8 % in Japan,^19^ 44.9 % in China,^20^ and 15.3 % in the United States demonstrating significant disparities and health inequities persist with little-to-no improvement in outcomes among preterm newborns in Ethiopia over the past 5 years or longer.^21^ More broadly, no significant improvements have been reported in survival of ELBW infants within LMICs across the last two decades.^18^ The minimal improvements in preterm mortality rates in these high-need, low resource settings highlight the importance of continued and sustained efforts to improve preterm neonatal care in Ethiopia and beyond. While the lack of decrement in preterm neonatal mortality rate may be due to inherent resource limitations, unbalanced patient-to-resource ratio, and higher nurse-to-patient ratio in LMICs, the lack of granular data investigating care practices and drivers of prematurity-related death within and between sites have not aided site-specific efforts to improve outcomes—especially for tertiary care NICUs such as SPHMMC where higher acuity patients may also translate into higher mortality despite higher levels of care.

We did analyze most common timeframes for neonatal deaths among premature neonates at SPHMMC as an additional factor to consider in determining priorities. The majority of admitted premature neonates died within the first 72 h after birth. In total, the first 72 h accounted for more than 65 % of prematurity-related deaths in the SPHMMC NICU (25 % within 24 h and 40.9 % within 24–72 h). This is consistent with an earlier U.S. analysis of extremely preterm infant outcomes born 22–28 + 6/7 weeks gestational age, where the median time of death was noted to be 3 days.^22^ Causes of death in this study were similar as well, with RDS and infection the leading two causes. However, a more recent cohort of extremely preterm infants in the U.S. found gradual shifting of risk of death beyond the first 3 days, with a bimodal peak at 7 days and at >28 days.^23^ It is possible that the gradual declining of early (within the first 72 h) death in these U.S.-based extremely preterm cohorts is a marker of overall improvements within in-hospital care practices and outcomes. If so, then ongoing close monitoring of timing of death may be an important trend to monitor at SPHMMC NICU and potentially other LMICs. We also noted a curious variation in prematurity-related deaths at SPHMMC by month, not directly correlated with the corresponding monthly number of admissions. Specifically, we noted that October appeared to have the lowest neonatal mortality (measured per 65 premature neonatal admissions to the NICU), which was a third of the mortality rate when compared with June. Seasonal variations in admissions have previously been described, although not clearly associated with mortality.^24^ Further investigation as to drivers of this variation may be warranted with monitoring if these are consistent, recurring patterns or spurious. It is also possible that these variations could be due to other factors other than seasonality such as staffing fluctuations that require further evaluation.

ACS is a well-evidenced method to reduce mortality secondary to prematurity.^25^ The WHO recommends all pregnant individuals who are highly likely to deliver between 24- to 34- weeks’ gestation receive ACS.^26^ Our study had an overall ACS exposure rate of 52.9 % of all preterm neonates admitted to the SPHMMC NICU. This value is higher than in 2021 when the reported ACS exposure rate of preterm neonates was 37.5 %,^27^ although still less than an ideal near-universal administration to mothers at risk of preterm birth. While the unadjusted analyses within this present study demonstrated higher raw mortality associated with ACS exposure, this finding is likely due to the fact that mothers of neonates with lower birthweights -a group with significantly higher mortality -were more likely to receive ACS. After adjusting for covariates and additional markers of severity of illness, there was no increased odds of mortality found between those exposed to ACS versus unexposed. With only half of eligible babies having been exposed to ACS, a more regimented and consistent approach is needed to ensure that all eligible babies benefit from ACS without bias of selecting only the sickest or smallest. Thus, this analysis points to an area of possible focus for SPHMMC as it relates to equitable exposure of all gestationally appropriate mothers to ACS given some suggestion of preferential administration to the smallest premature neonates potentially limiting appreciable improvements in mortality rates.

While prematurity is a well-documented leading contributor to neonatal death, data describing the specific diagnoses driving prematurity-related deaths in LMICs are limited. We believe our study is novel in the attempt to more granularly describe non-exclusive contributors to death among a large cohort of Ethiopian premature neonates. We found the three leading causes of mortality among admitted premature neonates were: RDS, sepsis (confirmed or presumed), and pulmonary hemorrhage. While RDS and sepsis have been previously documented as leading causes of mortality among preterm newborns in other settings,^17,24,28–31^ the finding of pulmonary hemorrhage as the third leading contributor to preterm mortality has been less well-described in the literature, albeit gaining some attention. Among a modern cohort of extremely preterm neonates 22–28 + 6/7 weeks gestation born in the U.S, pulmonary hemorrhage was a relatively frequent cause of death, being named as one of the top 5 leading causes of death and having once of the highest hazard ratios for death in the cohort.^23^ Impressively, pulmonary hemorrhage occurred in 1 in 4 deaths among this cohort of premature neonates admitted to SPHMMC NICU. These estimates are more than double what has been previously reported in a Brazilian cohort, citing estimates of 8–11 %.^32^ Another team from Botswana reported on all cases of pulmonary hemorrhage within their NICU from 2020–2021.^33^ While not restricted to only preterm newborns as in our study, the overall incidence of pulmonary hemorrhage was 4 % (54/1350 neonates) with an associated mortality rate of 53.7 % (29/54).^33^ However, a limitation of this and other LMIC studies is the reliance on the clinical diagnosis of pulmonary hemorrhage, considering the resource limitations in radiographical studies and autopsy. In our cohort, pulmonary hemorrhage diagnosis was made clinically by physicians caring for the neonates if bright red blood was observed coming through the oropharynx. Thus, it is possible that pulmonary hemorrhage was a symptom of a separate disorder or condition, such as the sequelae from birth asphyxia, coagulopathy, sepsis, or even mechanical issues such as limited humidity within the CPAP device. Interestingly all premature neonates with a diagnosis of pulmonary hemorrhage were intubated (n = 16) or on CPAP.

Additionally, the neonates in our cohort who died from pulmonary hemorrhage were less exposed to maternal chorioamnionitis, were more exposed to ACS, and were significantly more likely to be delivered by cesarean section but did not have any difference in respiratory support or vitamin K treatment, making it is unclear why these newborns ultimately died from pulmonary hemorrhage. Interestingly, a study conducted in the United States in the early 2000s evaluating very low birthweight neonates who died requiring cardiopulmonary resuscitation (CPR) to similar neonates who are age-matched controls reported a 17 % and 55 % pulmonary hemorrhage incidence within the control and CPR groups, respectively.^34^ In fact, while not reported for all cases, at least one case of pulmonary hemorrhage within the CPR group occurred prior to requiring CPR.^34^ What our study reports is similar to these findings from the United States over 2 decades ago.^34,35^ Ultimately, further studies are needed to evaluate whether the finding of pulmonary hemorrhage as being a leading contributor to preterm neonatal mortality is a true and valid finding and generalizable across other LMIC settings.

Given the findings in our study, it is critically important to further expand understanding related to the potential mechanisms related to the high rate of pulmonary hemorrhage affecting newborns in Ethiopia and other LMICs. For example, sepsis, asphyxia, and/or disseminate intravascular coagulopathy may all contribute to the high rate of pulmonary hemorrhage. Additionally, future studies are needed to explore the impact of surfactant, temperature dysregulation, ventilatory management, lack of humidification of respiratory support methods, and other factors on the development of pulmonary hemorrhage among small and/or sick newborns in LMICs are needed.

### 4.3. Research implications

Our study’s findings demonstrate that research and quality improvement initiatives are needed to promote high-quality care for SSNs in Ethiopia. Implementation and quality improvement research is an important need, to take evidence-based solutions that require specific training and resources, and implement them in low-resourced settings such as ACS administration to all eligible women, breastfeeding and kangaroo mother care.^26,36–39^ Additionally, research on the prevalence of pulmonary hemorrhage across other contexts in LMICs is needed to confirm if our study’s findings are generalizable. If so, research is needed to investigate the underlying reasons for the high rates of pulmonary hemorrhage within LMICs such as Ethiopia to ultimately determine ways to reduce the burden this condition imparts on the mortality rate. Similarly, while a trial has not been conducted, a randomized clinical trial evaluating the impact of surfactant (either via intubation, less invasive surfactant administration or aerosolized) on the treatment of pulmonary hemorrhage is critically needed as this intervention may be an important intervention to address RDS as the primary cause of death in LMICs. Inclusion of pulmonary hemorrhage as a balancing measure may be even more important in LMICs seeking to implement such an intervention.

### 4.4. Strengths and limitations

A strength of our study is evaluation of a contemporary cohort of preterm neonates born at a tertiary referral hospital in Ethiopia to provide data from 2022 to 2023. The data reflects the current clinical practices and diagnoses of death reflecting real-world data. However, our study also has significant limitations in that it is a retrospective study design with no standardized definitions of causes of death, including pulmonary hemorrhage as described above. Furthermore, the underlying causes of pulmonary hemorrhage are currently unknown and need further studies to help elucidate the contributors to the high rate of pulmonary hemorrhage in our population. Therefore, our study’s findings do not suggest causation but only associations between variables. Additionally, our study focuses on patients admitted to the NICU rather than all newborns delivered at SPHMMC. Any delivery room deaths or deaths prior to admission to the NICU are not captured in our dataset. Inclusion of these patients could have skewed the timing of death earlier (more in the first 24 h) and led to additional SSN outcomes being reported. Also, as a single-center study, this limits the generalizability of findings, which should be evaluated in other contexts and settings. Additionally, we are in the process of developing a developmental follow-up database to assess the long-term outcomes of children to ensure they not only survive but thrive. At this time, we are not able to comment on their long-term outcomes, although this is an aspiration for the future. Finally, there are no current standardized definitions for pulmonary hemorrhage within our setting other than blood coming from the oropharynx or endotracheal tube, which highlights that further standardization of a definition for pulmonary hemorrhage in neonates in LMICs is needed. The use of a clinical based definition, as involved in this study, could lead to classification bias.

## 5. Conclusion

Preterm mortality has not significantly decreased in the SPHMMC NICU for greater than 5 years. ELBW infants retain the highest mortality burden. RDS and sepsis (culture proven or presumed) remain the leading contributors to preterm infant mortality, with the highest risk of death within the first 72 h after birth. Surprisingly, the other leading clinical contributor to preterm mortality is pulmonary hemorrhage. While there exists a paucity of data on the incidence of pulmonary hemorrhage among preterm neonates in LMICs, there is emerging evidence of pulmonary hemorrhage as a significant harbinger of mortality in up to 50 % of diagnosed cases. We highlight the need to expand evaluation of the incidence and underlying risk factors for the development pulmonary hemorrhage in LMICs and the subsequent treatment of this life-threatening condition.

## Supplementary Material

1

2

Appendix A. Supplementary data

Supplementary data to this article can be found online at https://doi.org/10.1016/j.cegh.2025.102118.

## Figures and Tables

**Table 1:** Maternal and infant characteristics.

Maternal or Infant Factor		Mortality	No Mortality	p-value
Total Number of Infants		228	805	–
**Maternal Factors**				
Prenatal Care Visits		4 (4, 4)	4 (4, 4)	0.42
Multiple Gestation	Singleton	157/227 (69.2 %)	544/803 (67.7 %)	0.19
	Twins	63/227 (27.8 %)	246/803 (30.6 %)	
	Triplets or Higher	7/227 (3.1 %)	13/803 (1.6 %)	
Chorioamnionitis: Yes		3/154 (1.9 %)	32/497 (6.4 %)	**0.04**
Prenatal steroids: Full Course		100/225 (44.4 %)	280/796 (35.2 %)	**0.01**
Eclampsia: Yes		15/154 (9.7 %)	27/497 (5.4 %)	0.06
Pre-eclampsia: Yes		100/154 (64.9 %)	275/497 (55.3 %)	**0.04**
Chronic Diabetes: Yes		2/154 (1.3 %)	1/497 (0.2 %)	0.14
Gestational Diabetes: Yes		4/154 (2.6 %)	9/497 (1.8 %)	0.52
Mode of delivery: Cesarean Delivery		126/217 (58.1 %)	432/762 (56.7 %)	0.76
Age (years)		26.0 (23.2, 30.0)	26.0 (24.0, 30.0)	0.58
History of Preterm Birth: Yes		23/222 (10.4 %)	65/796 (8.2 %)	0.34
Maternal HIV: Yes		11/223 (4.9 %)	11/799 (1.4 % %)	0.75
Malnutrition: Yes		0/154 (0 %)	2/497 (0.4 %)	1.00
Residence: Urban		97/228 (42.5 %)	346/804 (43.0 %)	0.94
**Infants Characteristics**				
Gestational Age (weeks)		31 (29, 32)	34 (33, 35)	**<0.001**
Birthweight (g)		1200 (1000, 1485)	1850 (1500, 2100)	**<0.001**
Female sex		103/227 (45.4 %)	356/804 (44.3 %)	0.82
Intrauterine growth restriction (IUGR)		33/213 (15.5 %)	112/752 (14.9 %)	0.83
Apgar Score at 1 min		6 (5, 6)	7 (6, 7)	**<0.001**
Apgar Score at 5 min		7 (6, 8)	8 (7, 8)	**<0.001**
Inborn: Yes		192/226 (85.0 %)	782/804 (97.3 %)	0.76
Received Vitamin K		222/227 (97.8 %)	782/804 (97.3 %)	0.82
Any Respiratory Support During Hospitalization: Yes		221/227 (97.4 %)	625/801 (78.0 %)	**<0.001**
Type of Respiratory Support:				
Oxygen Cannula		7/227 (3.1 %)	182/801 (22.7 %)	**<0.001**
CPAP		212/227 (93.4 %)	496/801 (61.9 %)	
Ventilator		52/227 (22.9 %)	12/801 (1.5 %)	

All data presented as n/N (%) or median ± interquartile range. Fisher’s exact test and Mann-Whitney U tests used to assess categorical and numerical variables, respectively. Significant p-values bolded.

**Table 2:** Maternal and infant characteristics among inborns based on antenatal corticosteroid (ACS) exposure or lack thereof.

Maternal or Infant Factor		Any ACS	No ACS	p-value
Total Number of Infants		531	326	–
**Maternal Factors**				
Prenatal Care Visits		4 (4, 4)	4 (4, 4)	0.054
Multiple Gestation	Singleton	377/529 (71.3 %)	197/325 (60.6 %)	**<0.001**
	Twins	138/529 (26.1 %)	125/325 (38.5 %)	
	Triplets or Higher	14/529 (2.6 %)	3/325 (0.9 %)	
Chorioamnionitis: Yes		21/443 (4.7 %)	14/177 (7.9 %)	0.13
Eclampsia: Yes		26/443 (5.9 %)	14/177 (7.9 %)	0.37
Pre-eclampsia: Yes		291/443 (65.7 %)	74/177 (41.8 %)	**<0.001**
Chronic Diabetes: Yes		2/443 (0.5 %)	1/177 (0.6 %)	1.00
Gestational Diabetes: Yes		10/443 (2.3 %)	2/177 (1.1 %)	0.52
Mode of delivery: Cesarean Delivery		395/512 (77.1 %)	152/298 (51.0 %)	**<0.001**
Age (years)		26.0 (24.0, 30.0)	27.0 (24.0, 30.0)	0.47
History of Preterm Birth: Yes		44/524 (8.4 %)	26/322 (8.1 %)	0.90
Maternal HIV: Yes		2/526 (0.2 %)	8/324 (1.9 %)	0.06
Malnutrition: Yes		2/443 (0.5 %)	0/177 (0 %)	1.00
Residence: Urban		256/531 (48.2 %)	147/326 (45.1 %)	0.40
**Infants Characteristics**				
Gestational Age (weeks)		33 (31, 35)	34 (32, 35)	**<0.001**
Birthweight (g)		1600 (1300, 2000)	1900 (1500, 2200)	**<0.001**
Female sex		237/529 (44.8 %)	148/474 (31.2 %)	0.89
Intrauterine growth restriction (IUGR)		104/490 (21.2 %)	31/306 (10.1 %)	**<0.001**
Apgar Score at 1 min		7 (6, 7)	7 (6, 7)	0.32
Apgar Score at 5 min		8 (7, 8)	8 (7, 8)	0.37
Received Vitamin K		526/530 (99.2 %)	321/325 (98.8 %)	0.49
Any Respiratory Support During Hospitalization: Yes		483/530 (91.1 %)	237/325 (72.9 %)	**<0.001**
Type of Respiratory Support:				
Oxygen Cannula		92/530 (17.4 %)	64/325 (19.7 %)	0.07
Continuous Positive Airway Pressure (CPAP)		418/530 (78.9 %)	190/325 (58.5 %)	
Ventilator		34/530 (6.4 %)	16/325 (4.9 %)	
**Outcomes**				
In-Hospital Mortality		133/531 (25.0 %)	56/326 (17.2 %)	**0.008**

All data presented as n/N (%) or median ± interquartile range. Fisher’s exact test and Mann-Whitney U tests used to assess categorical and numerical variables, respectively. Significant p-values bolded.

**Table 3:** Maternal and infant characteristics comparing pulmonary hemorrhage as cause of death and other causes of death.

Maternal or Infant Factor		CAUSE OF DEATH: Pulmonary Hemorrhage	CAUSE OF DEATH: NOT Pulmonary Hemorrhage	p-value
Total Number of Infants		57	171	–
**Maternal Factors**				
Prenatal Care Visits		4 (4, 4)	4 (4, 4)	0.48
Multiple Gestation	Singleton	39/56 (69.6 %)	118/171 (69.0 %)	1.00
	Twins	16/56 (28.6 %)	47/171 (27.5 %)	
	Triplets or higher	1/56 (1.8 %)	6/171 (3.5 %)	
Chorioamnionitis: Yes		0/44 (0 %)	3/110 (2.7 %)	0.56
Prenatal steroids: Full Course		38/57 (66.7 %)	62/169 (36.7 %)	**<0.001**
Eclampsia: Yes		2/44 (4.5 %)	13/110 (11.8 %)	0.23
Pre-eclampsia: Yes		32/44 (72.7 %)	68/110 (61.8 %)	0.26
Chronic Diabetes: Yes		1/44 (2.3 %)	1/110 (0.9 %)	0.49
Gestational Diabetes: Yes		1/44 (2.3 %)	3/110 (2.7 %)	1.00
Mode of delivery: Cesarean Delivery		41/57 (71.9 %)	85/160 (53.1 %)	**0.02**
Age (years)		26 (24, 30)	26 (23, 30)	0.47
History of Preterm Birth: Yes		7/56 (12.5 %)	16/166 (9.6 %)	0.61
Maternal HIV: Yes		0/55 (0 %)	2/168 (1.2 %)	1.00
Malnutrition: Yes		0/44 (0 %)	0/110 (0 %)	1.00
Residence: Urban		25/57 (43.9 %)	72/171 (42.1 %)	0.88
**Infants Characteristics**				
Gestational Age (weeks)		31.0 (29.0, 32.0)	31.0 (29.0, 32.0)	0.63
Birthweight (g)		1100 (1000, 1450)	1200 (1000, 1470)	0.72
Female sex		25/56 (44.6 %)	78/171 (45.6 %)	1.00
Intrauterine growth restriction (IUGR): Yes		7/52 (13.5 %)	26/161 (16.1 %)	0.83
Apgar Score at 1 min		6 (5, 7)	6 (5, 6)	0.22
Apgar Score at 5 min		7 (6.5, 8)	7 (6, 8)	0.33
Received Vitamin K		54/57 (94.7 %)	168/170 (98.8 %)	0.10
Inborn: Yes		52/56 (92.9 %)	140/170 (82.4 %)	0.08
Any Respiratory Support During Hospitalization: Yes		55/56 (98.2 %)	166/171 (97.1 %)	1.00
Type of Respiratory Support:				
Oxygen Cannula		0/56 (0 %)	7/171 (4.1 %)	0.22
CPAP		52/56 (92.9 %)	160/171 (93.6 %)	
Ventilator		16/56 (28.6 %)	36/171 (21.1 %)	

All data presented as n/N (%) or median ± interquartile range. Fisher’s exact test and Mann-Whitney U tests used to assess categorical and numerical variables, respectively. Significant p-values bolded.

## Abbreviations:

ACS: Antenatal Corticosteroids
ACT: Antenatal Corticosteroids Trial
BW: Birth Weight
CI: Confidence Interval
ELBW: Extremely Low Birthweight
HIE: Hypoxic Ischemic Encephalopathy
IUGR: Intrauterine Growth Restriction
KCH: Kamuzu Central Hospital
KMC: Kangaroo Mother Care
LBW: Low Birth Weight
LMIC: Low- and Middle-Income Country
MDG: Millennium Development Goals
NICU: Neonatal Intensive Care Unit
NIH: National Institutes of Health
PTB: Preterm Birth
PNW: Post-Natal Ward
RDS: Respiratory Distress Syndrome
SDG: Sustainable Development Goals
SSN: Small and/or Sick Newborn
STROBE: Strengthening the Reporting of Observational Studies in Epidemiology
UN: United Nations
WHO: World Health Organization
