# Supplementary material for: A retrospective cohort study of premature neonatal mortality rates and contributing factors in a tertiary referral NICU in Addis Ababa, Ethiopia from 2022 to 2023
Source: Clin Epidemiol Glob Health. Author manuscript; Available in PMC 2025 Jul 31. (PMC12311812; doi:10.1016/j.cegh.2025.102118)
Supplement: 1 [file NIHMS2100128-supplement-1.docx]

**Supplementary Description of SPHMMC NICU Resources**

*SPHMMC NICU Resources*

The SPHMMC NICU has four separate subsections including the high dependency unit, preterm unit, subcritical unit, and the kangaroo mother care unit. The nurse-to-patient ratio is approximately 1:3 within all units except the kangaroo mother unit in which it is 1:8. Within the SPHMMC NICU, blood cultures are available to help diagnose culture-positive sepsis. Additionally, intravenous antibiotics, ventilatory support via conventional mechanical ventilators, and bubble continuous positive airway pressure (CPAP) are available for use in the NICU. The unit has 4 mechanical ventilators and 18 bubble CPAP devices. There are blenders for oxygen delivery in the NICU. Surfactant is not readily available unless the family can personally purchase surfactant from an outside vendor. Portable x-ray and echocardiograms are also available for use in the SPHMMC NICU.
