# Supplementary material for: A retrospective cohort study of premature neonatal mortality rates and contributing factors in a tertiary referral NICU in Addis Ababa, Ethiopia from 2022 to 2023
Source: Clin Epidemiol Glob Health. Author manuscript; Available in PMC 2025 Jul 31. (PMC12311812; doi:10.1016/j.cegh.2025.102118)
Supplement: 2 [file NIHMS2100128-supplement-2.docx]

**Supplemental Figures and Tables**


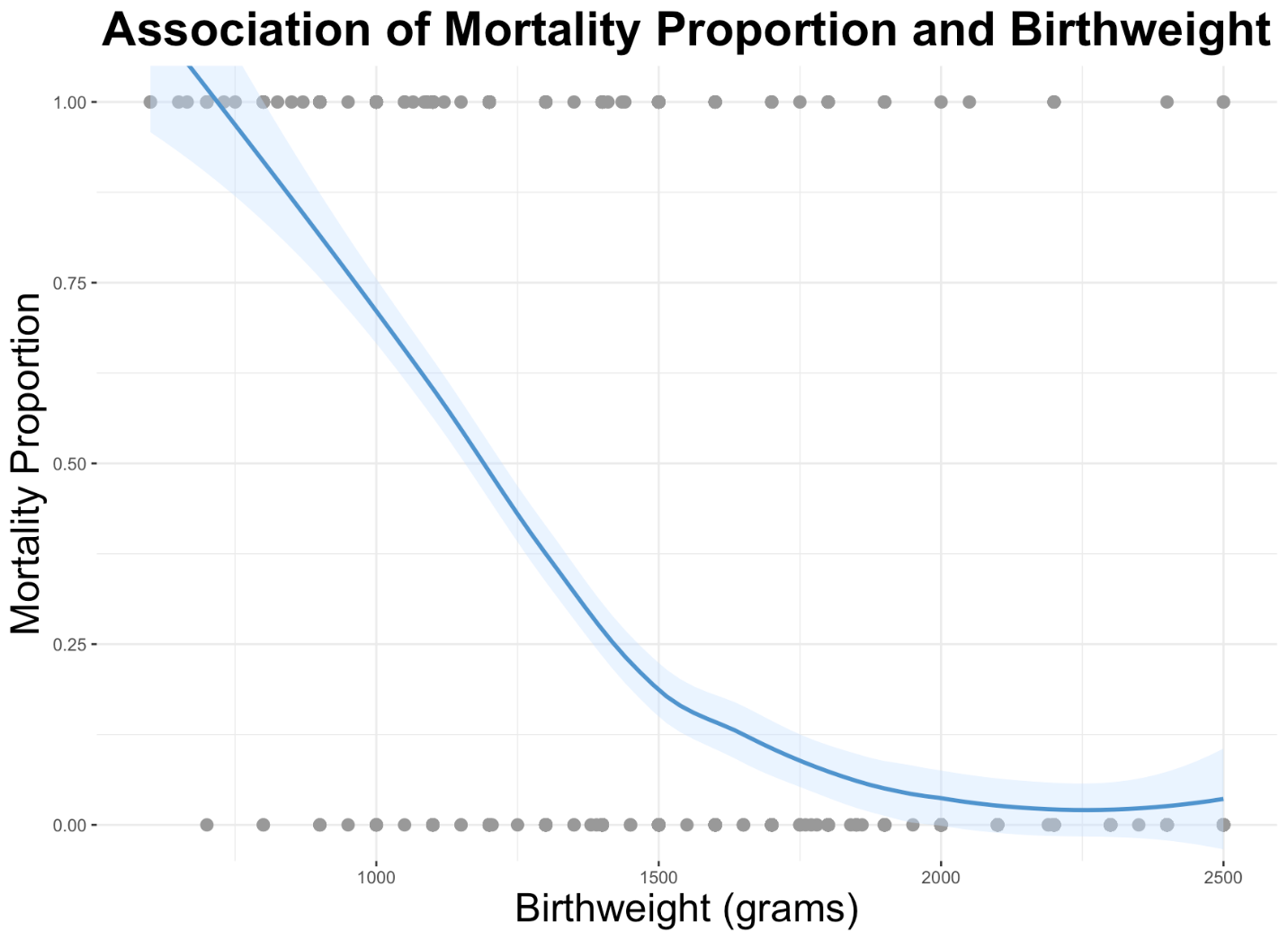


**Supplemental Figure 1.** Mortality proportion and association with birthweight. Unadjusted locally estimated scatterplot smoothing with 95% confidence shown.

**Supplemental Table 1: STROBE Statement—Checklist of items that should be included in reports of *cross-sectional studies***

|  | Item No | Recommendation |
| --- | --- | --- |
| **Title and abstract** | 1 | (*a*) Indicate the study’s design with a commonly used term in the title or the abstract |
|  |  | (*b*) Provide in the abstract an informative and balanced summary of what was done and what was found |
| Introduction | | |
| Background/rationale | 2 | Explain the scientific background and rationale for the investigation being reported |
| Objectives | 3 | State specific objectives, including any prespecified hypotheses |
| Methods | | |
| Study design | 4 | Present key elements of study design early in the paper |
| Setting | 5 | Describe the setting, locations, and relevant dates, including periods of recruitment, exposure, follow-up, and data collection |
| Participants | 6 | (*a*) Give the eligibility criteria, and the sources and methods of selection of participants |
| Variables | 7 | Clearly define all outcomes, exposures, predictors, potential confounders, and effect modifiers. Give diagnostic criteria, if applicable |
| Data sources/ measurement | 8* | For each variable of interest, give sources of data and details of methods of assessment (measurement). Describe comparability of assessment methods if there is more than one group |
| Bias | 9 | Describe any efforts to address potential sources of bias |
| Study size | 10 | Explain how the study size was arrived at |
| Quantitative variables | 11 | Explain how quantitative variables were handled in the analyses. If applicable, describe which groupings were chosen and why |
| Statistical methods | 12 | (*a*) Describe all statistical methods, including those used to control for confounding |
|  |  | (*b*) Describe any methods used to examine subgroups and interactions |
|  |  | (*c*) Explain how missing data were addressed |
|  |  | (*d*) If applicable, describe analytical methods taking account of sampling strategy |
|  |  | (*e*) Describe any sensitivity analyses |
| Results | | |
| Participants | 13* | (a) Report numbers of individuals at each stage of study—eg numbers potentially eligible, examined for eligibility, confirmed eligible, included in the study, completing follow-up, and analysed |
|  |  | (b) Give reasons for non-participation at each stage |
|  |  | (c) Consider use of a flow diagram |
| Descriptive data | 14* | (a) Give characteristics of study participants (eg demographic, clinical, social) and information on exposures and potential confounders |
|  |  | (b) Indicate number of participants with missing data for each variable of interest |
| Outcome data | 15* | Report numbers of outcome events or summary measures |
| Main results | 16 | (*a*) Give unadjusted estimates and, if applicable, confounder-adjusted estimates and their precision (eg, 95% confidence interval). Make clear which confounders were adjusted for and why they were included |
|  |  | (*b*) Report category boundaries when continuous variables were categorized |
|  |  | (*c*) If relevant, consider translating estimates of relative risk into absolute risk for a meaningful time period |
| Other analyses | 17 | Report other analyses done—eg analyses of subgroups and interactions, and sensitivity analyses |
| Discussion | | |
| Key results | 18 | Summarise key results with reference to study objectives |
| Limitations | 19 | Discuss limitations of the study, taking into account sources of potential bias or imprecision. Discuss both direction and magnitude of any potential bias |
| Interpretation | 20 | Give a cautious overall interpretation of results considering objectives, limitations, multiplicity of analyses, results from similar studies, and other relevant evidence |
| Generalisability | 21 | Discuss the generalisability (external validity) of the study results |
| Other information | | |
| Funding | 22 | Give the source of funding and the role of the funders for the present study and, if applicable, for the original study on which the present article is based |

*Give information separately for exposed and unexposed groups.

**Supplemental Table 2.** Frequency of cause of death by total number and subclassified by identified time frames.

| **COD (total number of times COD was listed)** | **Total number of times COD was listed** | **Percent of all babies who died (#/228)** | **Times listed ≤ 1 day** | **Percent of all babies who died ≤1 day (#/61)** | **Times listed days 2-7** | **Percent of all babies who died 2-7 days (#/107)** | **Times listed > 7 days** | **Percent of all babies who died >7 days (#/47)** | **unknown time** | **Percent of all babies who died with unknown time (#/13)** |
| --- | --- | --- | --- | --- | --- | --- | --- | --- | --- | --- |
| RDS | 159 | 69.7% | 49 | 80.3% | 81 | 75.7% | 20 | 42.6% | 9 | 69.2% |
| Dehydration or Shock | 3 | 1.3% | 1 | 1.6% | 1 | 0.9% | 1 | 2.1% | 0 | 0% |
| Cardiovascular | 17 | 7.5% | 2 | 3.3% | 10 | 9.3% | 3 | 6.4% | 2 | 15.4% |
| Congenital Heart Disease | 10 | 4.4% | 3 | 4.9% | 3 | 2.8% | 3 | 6.4% | 1 | 7.7% |
| Pulmonary Hemorrhage | 57 | 25.0% | 14 | 23.0% | 33 | 30.8% | 10 | 21.2% | 0 | 0% |
| NEC | 5 | 2.2% | 0 | 0% | 3 | 2.8% | 2 | 4.3% | 0 | 0% |
| Culture-positive Sepsis | 10 | 4.4% | 0 | 0% | 0 | 0% | 10 | 21.2% | 0 | 0% |
| Presumed Sepsis | 80 | 35.1% | 5 | 8.2% | 53 | 49.5% | 20 | 42.6% | 2 | 15.4% |
| Birth Asphyxia Complications | 8 | 3.5% | 5 | 8.2% | 2 | 1.9% | 1 | 2.1% | 0 | 0% |
| Meconium Aspiration Syndrome | 1 | 0.4% | 0 | 0% | 0 | 0% | 1 | 2.1% | 0 | 0% |
| Pneumonia | 0 | 0% | 0 | 0% | 0 | 0% | 0 | 0% | 0 | 0% |
| Meningitis | 0 | 0% | 0 | 0% | 0 | 0% | 0 | 0% | 0 | 0% |
| Congenital Anomalies | 3 | 1.3% | 0 | 0% | 1 | 0.9% | 2 | 4.3% | 3 | 23.1% |
| Multi Organ Failure | 13 | 5.7% | 2 | 3.3% | 5 | 4.7% | 3 | 6.4% | 3 | 23.1% |
| DIC | 3 | 1.3% | 0 | 0% | 2 | 1.9% | 1 | 2.1% | 0 | 0% |
| Apnea | 3 | 1.3% | 1 | 1.6% | 1 | 0.9% | 1 | 2.1% | 0 | 0% |
| IVH | 5 | 2.2% | 0 | 0% | 3 | 2.8% | 2 | 4.3% | 0 | 0% |
| Renal Failure | 1 | 0.4% | 0 | 0% | 0 | 0% | 1 | 2.1% | 0 | 0% |
| Aspiration | 8 | 3.5% | 0 | 0% | 0 | 0% | 8 | 17.0% | 0 | 0% |
| Other | 2 | 0.9% | 1 | 1.6% | 1 | 0.9% | 0 | 0% | 0 | 0% |

**Supplemental Table 3.** Number of babies that died by age of death and inborn status.

| **Age of death (days)** | **Number of babies that died** | **n/N (%) Inborn** |
| --- | --- | --- |
| 0 | 26 | 21/26 (81%) |
| 1 | 35 | 29/35 (83%) |
| 2 | 39 | 37/39 (95%) |
| 3 | 14 | 12/13 (92%) |
| 4 | 20 | 14/19 (74%) |
| 5 | 16 | 13/16 (81%) |
| 6 | 9 | 9/9 (100%) |
| 7 | 9 | 8/9 (89%) |
| 8-14 | 26 | 20/26 (77%) |
| 15-21 | 10 | 9/10 (90%) |
| 22-28 | 6 | 4/6 (67%) |
| >28 | 5 | 3/5 (60%) |
| Total | 215 | 179/213 (84%) |


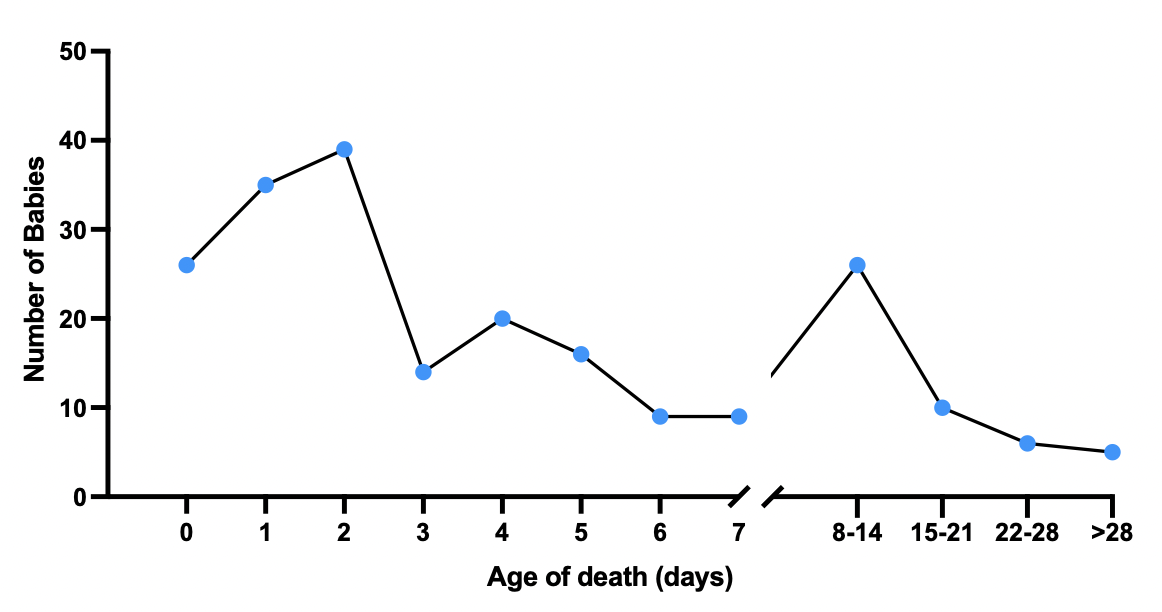


**Supplemental Figure 2.** Number of babies that died by of age of death.


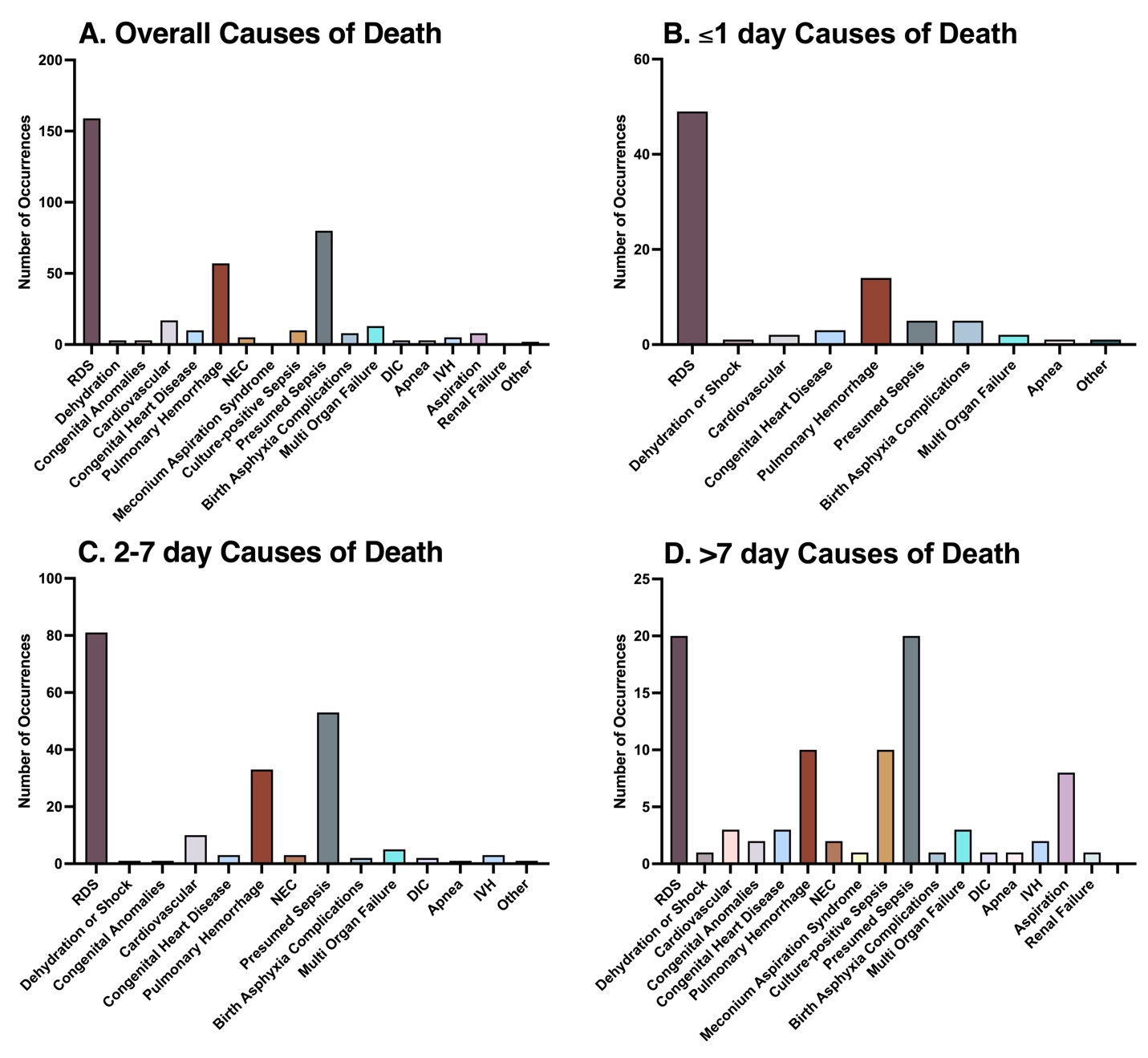


**Supplemental Figure 3**: Bar chart of the causes of death of premature newborns at SPHMMC NICU per chronological time after birth.


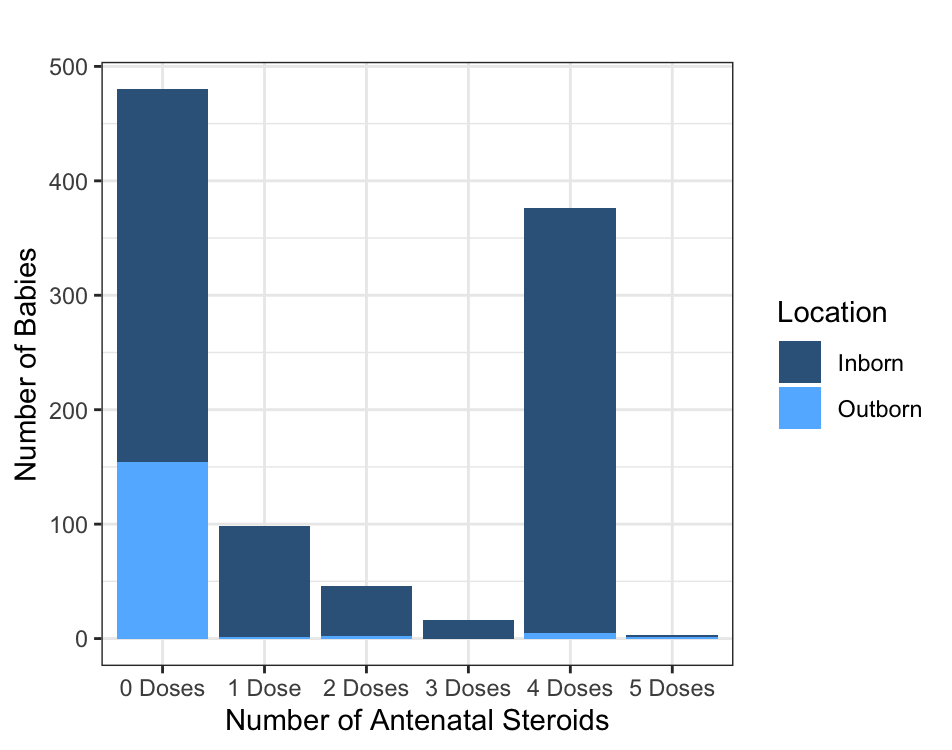


**Supplemental Figure 4.** Number of babies per antenatal steroid course. 481 babies did not receive antenatal steroids (326 inborn, 154 outborn, 1 unknown), 98 babies received 1 dose (97 inborn, 1 unknown), 46 babies received 2 doses (44 inborn, 2 outborn), 16 babies received 3 doses (all inborn), 378 babies received 4 doses (371 inborn, 5 outborn), 3 babies received 5 doses (2 inborn, 1 outborn). Steroids were unknown for 11 babies, and birth location was unknown for 4 babies.

**Supplemental Table 4:** Antenatal steroid exposure and association with in-hospital mortality among inborns by ACS full course, partial course, or lack of exposure.

| **Event** | **Odds Ratio (95%CI)** | **p-value** | **Adjusted* Odds Ratio (95%CI)** | **Adjusted p-value** |
| --- | --- | --- | --- | --- |
| **In-Hospital Mortality** | | | | |
| Full Course (4+ doses) | 1.69  (1.17, 2.45) | **0.005** | 1.19  (0.68, 2.07) | 0.54 |
| Partial Course (1-3 doses) | 1.43  (0.90, 2.30) | 0.13 | 0.89  (0.45, 1.77) | 0.75 |
| No Antenatal Steroids | Reference | - | Reference | - |

Only babies born at St. Paul’s were included in analyses. Logistic regression models were used to examine the association of antenatal steroids with mortality.

*Model adjusted for gestational age, IUGR status, mode of delivery, pre-eclampsia, multiple gestation, and if baby required respiratory support during hospitalization. Significant p-values bolded.

**Supplemental Table 5:** Frequency of cause of death listed with pulmonary hemorrhage.

|  | **Total number of times COD was listed with PH** |
| --- | --- |
| RDS | 43 |
| Dehydration or Shock | 0 |
| Cardiovascular | 3 |
| Congenital Heart Disease | 0 |
| NEC | 1 |
| Culture-positive Sepsis | 0 |
| Presumed Sepsis | 15 |
| Birth Asphyxia Complications | 1 |
| Meconium Aspiration Syndrome | 0 |
| Pneumonia | 0 |
| Meningitis | 0 |
| Congenital Anomalies | 0 |
| Multi Organ Failure | 2 |
| DIC | 1 |
| Apnea | 1 |
| IVH | 0 |
| Renal Failure | 1 |
| Aspiration | 0 |
| Other | 0 |
| Prematurity | 43 |
